# Case Report and Literature Review: Post-Arthroscopy Pneumothorax with Anterior Decompression

**DOI:** 10.5811/cpcem.2020.8.48618

**Published:** 2020-10-09

**Authors:** Marc A. Cassone, Kristin L. Kish, Jordan R. Nester, Lisa M. Hoffman

**Affiliations:** * Geisinger Medical Center, Department of Emergency Medicine, Danville, Pennsylvania; † Geisinger Medical Center, Department of Orthopedic Surgery, Danville, Pennsylvania

**Keywords:** Shoulder surgery complication, subcutaneous emphysema, chest tube, thoracostomy

## Abstract

**Introduction:**

Emergency providers should recognize that pneumothorax is a rare but serious complication of shoulder arthroscopy that may require a unique approach to decompression.

**Case Report:**

We present a case of a 60-year-old female who presented to the emergency department with right-sided facial swelling, voice change, and shortness of breath three hours after an elective arthroscopic right rotator-cuff repair and was noted to have a right-sided pneumothorax. We also describe a potential novel approach to chest tube decompression that maintains shoulder adduction in patients with recently repaired rotator cuffs.

**Conclusion:**

Although most cases of post-arthroscopy pneumothoraces are reported in patients who received regional anesthesia or have underlying lung pathology, it can occur in lower-risk patients as was demonstrated in our case. We also suggest considering an alternative anterior approach between the midclavicular and anterior axillary lines for chest decompression in select patients when a traditional approach is less ideal due to the need to maintain shoulder immobilization postoperatively.

## INTRODUCTION

Over the past few decades shoulder arthroscopy has become an increasingly common technique to treat shoulder pathology. Proponents cite a decreased complication rate when compared to open procedures.^1^ Others report not necessarily a lower complication rate but a different set of complications altogether including neurovascular injuries, infection, venous thromboembolic events, pneumothoraces, and soft tissue and neuropraxic injuries due to positioning.^2^ We present a case of a 60-year-old female who presented to the emergency department (ED) with right-sided facial swelling, voice change, and shortness of breath three hours after an elective arthroscopic, right rotator cuff repair and was noted to have a right-sided pneumothorax. Patients undergoing shoulder repair often require immobilization to ensure proper alignment and healing that may limit anatomic placement of chest thoracostomy tubes. We suggest a possible variation of the traditional approach that may be considered in these cases.

## CASE REPORT

**Image 1:**
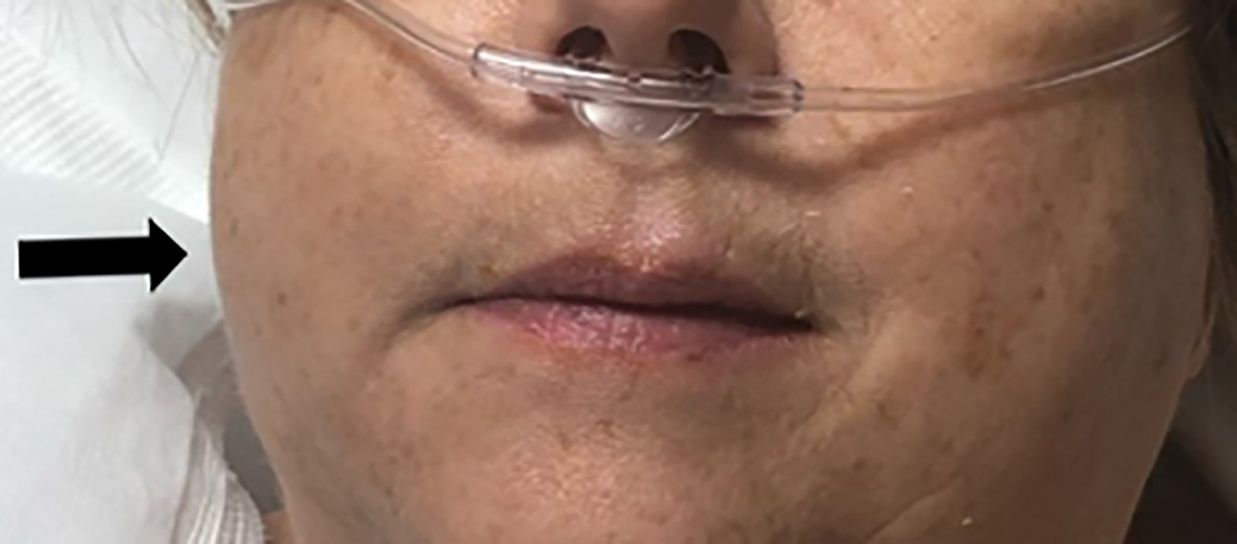
Right-sided facial swelling and crepitus noted several hours postoperatively. Patient also reported voice change and dyspnea. Facial swelling denoted by arrow.

A 60-year-old female presented to the ED with right-sided facial swelling, voice change, and shortness of breath three hours after an elective, arthroscopic, right rotator cuff repair. The outpatient surgery comprised a subacromial decompression, major glenohumeral joint debridement, and rotator cuff repair for right shoulder chronic impingement syndrome, intra-articular biceps tear, and full thickness rotator cuff tear. The surgery was performed in the lateral decubitus position under general anesthesia and without regional nerve block. The anesthesia report was reviewed and showed no major complications or episodes of hypotension or desaturation. The patient reported feeling well postoperatively; however, three hours later she developed right-sided face and neck swelling (Image 1), voice changes, and dyspnea, and thus presented to the ED for further evaluation.

**Image 2:**
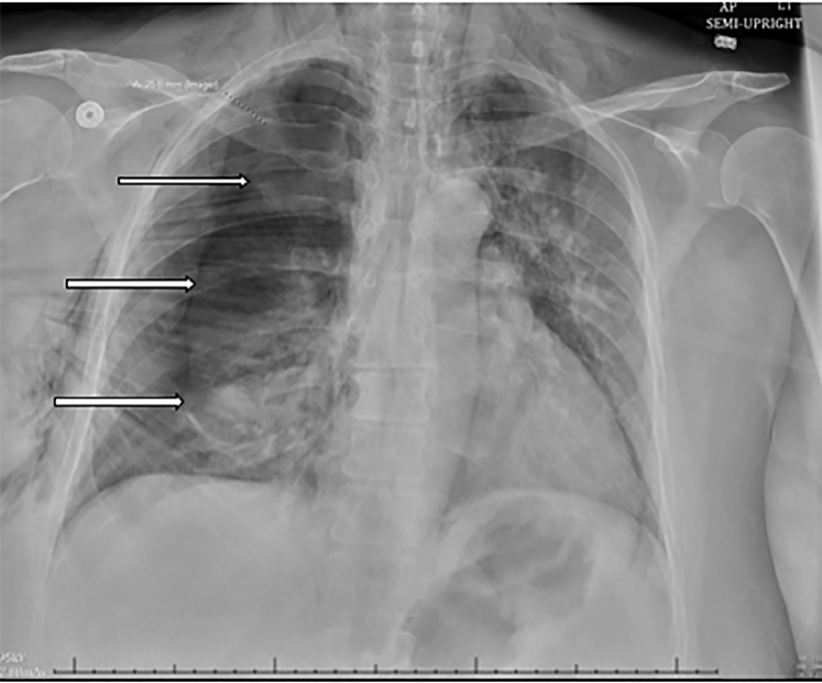
A 70% large right-sided pneumothorax (arrows) with left-sided mediastinal shift and extensive subcutaneous emphysema (“gingko leaf sign”).

The patient denied significant past medical history including any history of smoking or underlying lung or connective tissue disease. On arrival, her vitals were heart rate 92 beats per minute, blood pressure 135/80 millimeters mercury, respiratory rate 18 breaths per minute, and pulse oximetry was 96% on room air. Her physical exam was notable for predominately right-sided facial swelling, diminished right-sided lung sounds, and crepitus of the right neck, face, and chest. A chest radiograph showed a right-sided pneumothorax of approximately 70% with mediastinal shift and extensive subcutaneous emphysema (Image 2).

**Image 3:**
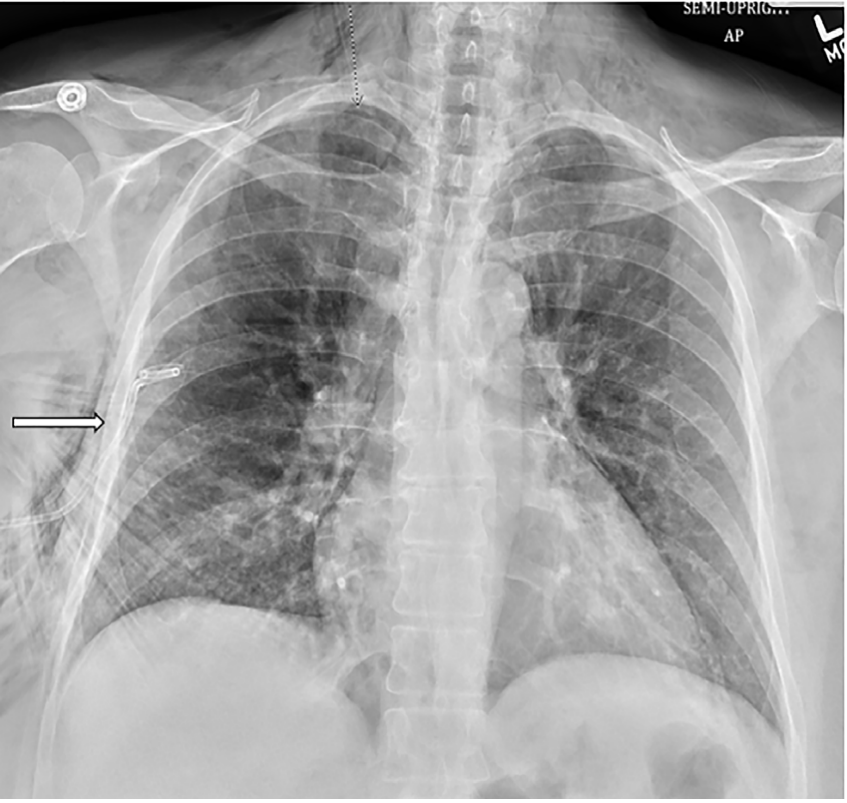
Successful lung re-expansion after right-sided pigtail catheter (arrow) was placed using a more anterior approach. Note improved mediastinal shift.

A pigtail catheter-type chest drain was placed in the fifth intercoastal space between the right midclavicular and anterior axillary line so as to not abduct or displace the patient’s shoulder given her recent rotator cuff repair. Successful expansion of the lung was noted (Image 3).

On the third day of admission, the chest tube was removed and the patient was discharged home without further complications. At her two-week follow-up, her shoulder was healing well and she had no significant sequelae from her pneumothorax.

## DISCUSSION

Only a handful of case reports and case series describe an association between arthroscopic shoulder surgery and postoperative pneumothorax^3^–^8^; however, cases may be under-reported. It has been postulated that these may be related to preoperative regional anesthesia (notably the interscalene brachial plexus block),^5^,^6^,^8^ intubation and related airway trauma,^3^,^9^ as well as injury to the parietal pleura from laparoscopy including continuous positive pressure-driven pump infusion in the joint space or intra-articular shaving.^3^,^7^ Patient positioning, including the beach chair vs lateral decubitus, has also been posited as an increased risk for pneumothorax.^7^ Of the cases reported, the majority had underlying lung disease (chronic obstructive pulmonary disease, asthma, chronic tobacco abuse).^5^,^6^ For cases not related to regional anesthesia, all those reported occurred within 1–24 hours of surgery.^3^–^8^ Hospital courses were largely unremarkable, and most patients were discharged within two to five days of presentation^3^–^8^ with the most common complication reported being chest tube leak.^4^,^5^ The vast majority of reported cases presented with either face, neck, or chest subcutaneous air on exam or imaging,^3^–^8^ and only one episode of hypotension from tension pneumothorax was reported.^3^

CPC-EM Capsule

What do we already know about this clinical entity?

*Pneumothoraces have been reported as a possible complication of shoulder arthroscopy*.

What makes this presentation of disease reportable?

*We present a case of a rare complication of a common procedure and suggest a less common procedural approach to treating pneumothorax*.

What is the major learning point?

*Pneumothorax is a rare but serious complication of shoulder arthroscopy that may require a unique approach to decompression*.

How might this improve emergency medicine practice?

*This variation to traditional pneumothorax decompression technique could help avoid re-injury of the rotator-cuff repair site*.

Emergency providers have traditionally placed tube thoracostomy drains at the fourth or fifth intercostal space at the midaxillary or anterior axillary line^10^ to allow for effective decompression and to avoid injury to mediastinal, cervical, and sub-diaphragmatic structures. However, to preserve the shoulder immobilization in the adducted position, the providers in this case placed the pigtail catheter at the fifth intercostal space between the midclavicular and anterior axillary line. No neurovascular injuries or injuries to mediastinal structures were noted during the procedure or in the subsequent days prior to removal. While decompression of the pneumothorax (especially with signs of mediastinal shift) should supersede the need to keep postoperative immobilization, we suggest this as a variation to the approach of the traditional technique to help avoid re-injury of the rotator cuff repair site. Complications of this more anterior approach could include injury to the lateral thoracic artery, long thoracic nerve, and local lymphatics; providers should ensure proper needle insertion technique and note local neurovascular findings before and after decompression. However, even using the traditional approach between the midaxillary or anterior axillary line can lead to local neurovascular injury.^11^

Limitations to this new approach include left-sided pneumothorax (given proximity to mediastinal structures), obese patients, large area of breast tissue, or use of larger caliber chest tubes. Placing the pigtail catheter at the second intercostal space midclavicular line is a viable alternative in these cases^12^; however, this technique is not without complications in correct placement,^13^ efficacy,^14^ or local vascular injury.^15^ Providers should evaluate patients on a case-by-case basis to determine the best approach for decompression. The variation on a traditional approach described in this case offers an alternative option in postarthroscopy patients; however, the authors recommend further studies to confirm safety and efficacy compared to other techniques.

## CONCLUSION

Emergency providers should recognize pneumothorax as a rare but serious complication of shoulder arthroscopy. Although most cases reported are in patients who received regional anesthesia or have underlying lung pathology, it can occur in lower-risk patients as was demonstrated in our case. Patients may present with classical findings of pneumothorax including dyspnea, decreased breath sounds, and signs of tension including mediastinal shift. Postarthroscopy pneumothoraces nearly always present with crepitus on exam or subcutaneous air on imaging. The majority of these cases tolerate decompression without issues and patients are discharged within several days without complications. Providers may consider follow-up computed tomography imaging in patients without prior lung disease to assess for underlying lung disease or structural pathology including blebs. We also suggest considering an alternative anterior approach between the midclavicular and anterior axillary lines for chest decompression in select patients when a traditional approach is less ideal due to the need to maintain shoulder immobilization postoperatively; however, more research is needed to confirm safety and efficacy.

## References

[1] Weber SC, Abrams JS, Nottage WM (2002). Complications associated with arthroscopic shoulder surgery. Arthroscopy.

[2] Moen T, Rudolph G, Caswell K (2014). Complications of shoulder arthroscopy. J Am Acad Orthop Surg.

[3] Bamps S, Renson D, Nijs S (2016). Pneumothorax after shoulder arthroscopy: a rare but life-threatening complication. J Orthop Case Rep.

[4] Asghar S, Azam M, Gjeka R (2015). Spontaneous pneumothorax with extensive subcutaneous emphysema: a rare complication of shoulder arthroscopy. Chest.

[5] Leander-Olsson O, Borglun-Hemph A, Jakobsson J (2016). Pneumothorax following shoulder arthroscopy under combined regional and general anesthesia: a case report. Int Jour Case Rep Imag.

[6] Calvisi V, Lupparelli S, Rossetti S (2009). Subcutaneous emphysema and pneumomediastinum following shoulder arthroscopy with brachial plexus block: a case report and review of the literature. Arch Orthop Trauma Surg.

[7] Lee HC, Dewan N, Crosby L (1992). Subcutaneous emphysema, pneumomediastinum, and potentially life-threatening tension pneumothorax: pulmonary complications from arthroscopic shoulder decompression. Chest.

[8] Tandon S, Taxak S, Gupta KB (1998). Pneumomediastinum: a rare complication of brachial plexus block. Indian J Chest Dis Allied Sci.

[9] Marty-Ané CH, Picard E, Jonquet O (1995). Membranous tracheal rupture after endotracheal intubation. Ann Thorac Surg.

[10] Margolis AM, Kirsch KD, Hedges JR, Roberts JR (2019). Tube thoracostomy. Roberts and Hedges’ Clinical Procedures in Emergency Medicine and Acute Care.

[11] Wraight WM, Tweedie DJ, Parkin IG (2005). Neurovascular anatomy and variation in the fourth, fifth, and sixth intercostal spaces in the mid-axillary line: a cadaveric study in respect of chest drain insertion. Clin Anat.

[12] Gammie JS, Banks MC, Fuhrman CR (1999). The pigtail catheter for pleural drainage: a less invasive alternative to tube thoracostomy. JSLS.

[13] Ferrie EP, Collum N, McGovern S (2005). The right place in the right space? Awareness of site for needle thoracentesis. Emerg Med J.

[14] Inaba K, Ives C, McClure K (2012). Radiologic evaluation of alternative sites for needle decompression for tension pneumothorax. Arch Surg.

[15] Rawlins R, Brown KM, Carr CS (2003). Life threatening haemorrhage after anterior needle aspiration of pneumothoraces. A role of lateral needle aspiration in emergency decompression of spontaneous pneumothorax. Emerg Med J.

